# CRISPR Gene Editing of Human Primary NK and T Cells for Cancer Immunotherapy

**DOI:** 10.3389/fonc.2022.834002

**Published:** 2022-04-05

**Authors:** Ezgi Elmas, Noushin Saljoughian, Marcelo de Souza Fernandes Pereira, Brian P. Tullius, Kinnari Sorathia, Robin J. Nakkula, Dean A. Lee, Meisam Naeimi Kararoudi

**Affiliations:** ^1^Molecular, Cellular and Developmental Biology Graduate Program, The Ohio State University, Columbus, OH, United States; ^2^Center for Childhood Cancer and Blood Diseases, Abigail Wexner Research Institute at Nationwide Children’s Hospital, Columbus, OH, United States; ^3^CRISPR/Gene Editing Core, Abigail Wexner Research Institute at Nationwide Children’s Hospital, Columbus, OH, United States; ^4^Pediatric Cellular Therapy, AdventHealth for Children, Orlando, FL, United States; ^5^Department of Pediatrics, The Ohio State University, Columbus, OH, United States

**Keywords:** NK cells, CRISPR, T cell, immunotherapy, off-target analysis, CRISPR screening, CAR-T cells, CAR-NK cell

## Abstract

Antitumor activity of immune cells such as T cells and NK cells has made them auspicious therapeutic regimens for adaptive cancer immunotherapy. Enhancing their cytotoxic effects against malignancies and overcoming their suppression in tumor microenvironment (TME) may improve their efficacy to treat cancers. Clustered, regularly interspaced short palindromic repeats (CRISPR) genome editing has become one of the most popular tools to enhance immune cell antitumor activity. In this review we highlight applications and practicability of CRISPR/Cas9 gene editing and engineering strategies for cancer immunotherapy. In addition, we have reviewed several approaches to study CRISPR off-target effects.

## Introduction

In recent years, adoptive T cell and NK cell therapies and immune checkpoint blockades have been successfully used in the clinic to improve immunotherapy for cancer. Immunotherapies with T and NK cells aim to overcome tumor-mediated immunosuppression and augment immunity against cancer (1–3). Adoptive T cell cancer immunotherapies comprehend tumor-infiltrating lymphocytes (TILs), transgenic T cell receptor (TCR)- T cell and chimeric antigen receptors (CAR)-T cell therapies (1). NK cell immunotherapies with cytokine stimulation, antibodies, and gene CAR-NK cells have been studied to overcome immunosuppression in cancers (2, 4). Although advancement in immunotherapy has been significant and durable, most cancer patients fail to respond to immunotherapy due to resistant tumor nature. Thus, we urgently need to find novel immunotherapies for cancer patients.

CRISPR/Cas9 gene-editing technology application has been widely studied and used in cancer immunotherapy research (5, 6). CRISPR method offers precise and powerful gene-editing efficiency in cancer and immunotherapy research. It has been used to identify essential genes as immune checkpoint targets, generate CAR-T and CAR-NK cells, construct TCR, understand signaling pathways, and screen for new druggable targets in immunotherapy (1, 7–10).

In this review, we describe the fundamentals of CRISPR gene editing in primary human T cells and NK cells. In addition, we highlight the applications of CRISPR/Cas9 technology in engineered T cells and NK cells and how it improves the immune cell function against cancers. Furthermore, several approaches to study off-target effects of CRISPR has been discussed.

## CRISPR Gene Editing

CRISPR are classes of repeated DNA sequences that act in coordination with CRISPR-associated (Cas) genes to devote bacterial and archaeal immunity against foreign raider phages and plasmid DNA (11). This system has been tested in several human cells including primary immune cells such as T-cells and NK cells. CRISPR consists of three elements: tracer-RNA, crispr-RNA (complementary to the target gene) and the Cas nuclease protein (12). Recognition of the target gene by guideRNA (Tracer-RNA + crispr-RNA) bound to Cas protein results in double stranded break (DSB) (5, 13, 14). DSBs can be repaired by one of the two highly conserved competing repair mechanisms, named as nonhomologous end-joining (NHEJ) or homology-directed repair (HDR) pathways (15). NHEJ results in insertion/deletion (indel) of nucleotides at the Cas9 targeting site and causes a frame shift in coding region and introduces gene knock-out (15). On the other hand, HDR is essential for insertion of a transgene such as a DNA template encoding a CAR into the Cas9 targeting site through homology repairs when homologous arms for the flanking region of Cas9 targeting site are provided in the DNA template (5). The best approach to deliver CRISPR elements and the DNA template depends on the target tissue or cell, packaging capacity, immunotoxicity, tropism, and integration site (5). Viral delivery has been widely used for human cells. Some of them are non-integrative, like the adeno-associated viruses (AAV) and adenoviruses (AdV), while some are integrative, such as Retroviridae family (MLV; murine leukemia virus or HIV; human immunodeficiency virus) (16, 17). Stable expression of the CRISPR in human primary cells is challenging due to the activation of anti-viral activity of the cells especially in NK cells and expressing a big protein like Cas9 results in low efficiency (18, 19). Therefore, delivery of pre-transcribed gRNA and pre-translated Cas9 as Cas9/Ribonucleoprotein (Cas9/RNP) has been favorable in immune cells (20, 21). Generation CAR expressing immune cells by site-directed gene insertion has been shown to be successful in both NK and T-cells. In this approach the DNA encoding a CAR is delivered as an HDR template by AAV vectors following electroporation of Cas9/RNP (22, 23). Providing optimal homology arms for Cas9-targeting site in the HDR template would be challenging as AAV has a small packaging capacity (less than 5 kb) (24). We have shown that a minimum of 300bp homology arms is required for high efficiency of the transgene integration into the Cas9 targeting site (23).

## Introduction to T Cells and Their Role in Cancer Immunotherapy

**Figure 1:**
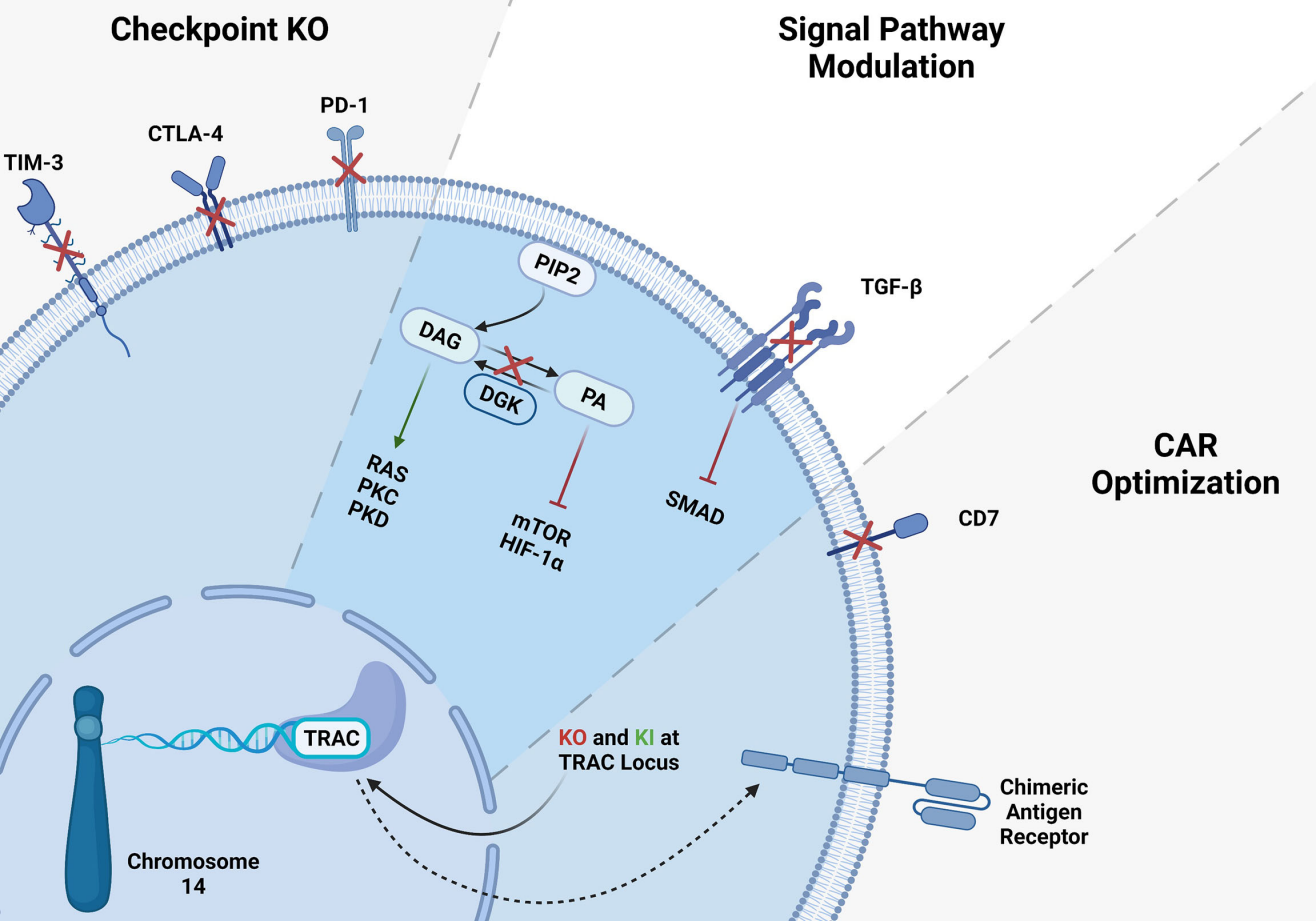
CRISPR gene editing in T-cells. Several gene KO and KI have been tested in T-cells, here we summarized the targeted genes. T cell checkpoint inhibitory receptor KO such as TIM3, CTLA-4 and PD-1 KO resulted in higher antitumor activity of T-cells. CAR-T cell signaling modulation *via* inhibition of immunosuppressive TGF-β signaling showed significant improvement of CAR-T cells. Integration of CAR-T in TRAC locus may solve the mentioned problems with allogeneic CAR-T therapies.

T cells are one of the most prominent components of the adaptive immune response. They can be distinguished from other lymphocytes by possessing TCR on their cell surface. T cells are developed in the thymus, and they recognize the antigen peptides presented by major histocompatibility complexes (MHC) class I and class II. T cells have two major CD8+ and CD4+ subtypes. CD8+ T cell refers to killer T cells, and CD4+ T cell refers to helper T cells. CD8+ killer T cells are involved in directly eradicating the virally infected cells as well as cancer cells. Even though T cells incredibly work and eliminate the most frustrating cancers, cancer remains one of the most devastating diseases globally and the leading cause of death. Conventional treatment options such as chemotherapy, radiotherapy and surgery have not been very effective in treating cancers. Recently, cell-based therapies, checkpoint blockades, cancer vaccines, oncolytic viruses and other forms of immunotherapies have shown promising clinical outcomes. T cell-based therapies are among the most efficient immunotherapies for cancer patients due to their eminent clinical efficacy (25). These new immunotherapies rely on the ability of T cells to eradicate tumors (26, 27). To enhance their antitumor activity and specificity, great interest in CAR- T cells has been evolved and have been used to treat hematologic malignancies and solid tumors. In autologous CAR-T cell-based therapies, the patient’s own T cells are genetically engineered to express a single-chain CAR which includes an antibody extracellular binding domain that recognizes a tumor cell surface antigen. Tumor antigen is recognized by extracellular domain of the CAR. Signaling activation is achieved by both costimulatory molecule such as CD27, CD28, 41BB and CD3zeta which contains ITAM motives (28). Thus, the engineered CAR-T cells can bind to tumor antigens and lyse the tumor cells independently from MHC, whereas normal T cells require TCR binding to an MHC class peptide antigen for their activation (19). Although CAR-T cell immunotherapies have been shown to be the most promising FDA approved cell based treatments, several challenges remain to be tackled (29). There has been some severe adverse events associated with CAR T cell toxicities (30–37). For example, most of the clinical trials use autologous T cells isolated from patients’ blood. This results in cell manufacturing failures from the early phase of the trial, due to low T cell quality and lymphocyte counts in some of the heavily treated patients (38). Manufacturing of autologous CAR T cell is a time-consuming process, therefore delaying the treatment in patients (33, 34). Additionally, when apheresis product is used for CAR-T cell production, sometimes failure in the process causes unsuccessful CAR-T cell manufacturing and poor response to treatment (30, 39–41). To overcome the problems related to autologous CAR-T cells, allogeneic CAR-T cell therapies has become alternative to autologous CAR-T cells (42–44). However, allogeneic CAR-T cell recognize and attack the recipient’s tissues causing graft-versus-host disease (GvHD) therefore limiting their use in the clinic (45–48). In addition to that, in both autologous and allogeneic CAR-T cells, side effects such as cytokine release syndrome (CRS) and neurologic toxicity in patients remains a challenge to overcome (34–37, 49–55). Efforts in gene-editing technologies such as CRISPR gene editing aid as a potential tool for overcoming the barriers in CAR-T immunotherapies (**Figure 1**) (27, 38, 56–62).

## Examples of CRISPR Edited T Cells

Genome editing technologies facilitate remarkable, highly efficient, and specifically targeted genomic modifications. CRISPR/Cas9 technology has been the most practical and efficient gene-editing method among other strategies for editing the T cells (63–66). Producing off-the-shelf universal CAR- T cells, overcoming T cell exhaustion, and suppressive TME become significant obstacles which CRISPR can be a suitable tool to tackle those issues (**Figure 1**) (44, 63). Several groups have reported successful gene editing of T-cells using Cas9/RNP (66, 67). Electroporation of Cas9/RNP to edit T-cells has been very efficient and been successfully used in the clinic to treat cancers (68). To solve the limitations of antigen-specific and HLA-matched T cells and generate universal allogeneic CAR-T cells, genetically engineered TCR complexes were developed for immune therapy. Targeted gene editing in T cells has major advantages over lentiviral transduction platforms. For example, lentiviral transduction of TCR leads to variable transgene copy numbers and untargeted transgene integration and therefore initiates variable TCR expression and functionality. Oppositely, TCR editing with CRISPR/Cas9 allows high-efficient gene targeting and avoids random integration (63, 64). CRISPR/Cas9 strategy has also been used to target PD-1, CTLA-4, LAG-3, and TIM-3 inhibitory molecules to overcome tumor mediated immune suppression and enhance CAR-T cell function (22, 69, 70). It also has been shown that diacylglycerol kinase (DGK) CRISPR-Cas9 KO improves the anti-tumor activity of CAR-T cells (71). TGF-β receptor II (TGFBR2) KO with CRISPR/Cas9 was also shown to reduce CAR-T exhaustion and increase the anti-tumor activity of CAR-T cells (72). Inhibition of CD7 and TRAC using CRISPR/Cas9 enhances CAR-T cell-killing activity and prevents fratricide against T-ALL. Sterner et al. (73) showed that CRISPR/cas9 KO of granulocyte-macrophage colony-stimulating factor (GM-CSF) decreased the side effects like cytokine release syndrome and neuroinflammation of CAR-T cell therapies and also improved the CAR-T cell anti-tumor activity *in-vivo* (73). CRISPR/Cas9 gene not only used for KO, it has been also utilized for gene insertion of exogenous DNAs. Site directed gene knock-in (KI) has improved CAR-T cell antitumor efficiency (74, 75). Several approaches have been developed to deliver the DNA template encoding CARs. Schumann et al. introduced a HDR template into the CXCR4 gene locus by electroporation of a plasmid DNA and Cas9/RNP, and demonstrated successful site directed KI (75). Moreover, insertion of CD19 specific CAR expressing DNA into the TRAC locus has been achieved with the CRISPR/Cas9 method and improved CAR-T cell efficiency. To generate these cells, T cells were electroporated with Cas9 mRNA and gRNA. Next, the HDR template encoding CD19 CAR was delivered to the cells *via* AAV6 transduction (60). In the T cell engineering era, insertions or deletions of short sequences with CRISPR/Cas9 technology have been very effective, precise, and routinely used. However, it has also been possible to KI longer sequences using ssDNA inserts called the Easi-CRISPR method with high efficiency (74). Cas9 is the most used endonuclease protein in CRISPR systems, but other Cas proteins such as Cas12 or Cpf1 is also used to generated CAR-T cells when combined with AAV gene delivery (22). To generate CAR-T cells with simultaneous KO of checkpoints and knock-in of double CARs, a method called KIKO has been developed. This method uses AAV-Cpf1 to generate KO and double knock-in KIKO-CAR-T cells (22, 76).

## Introduction to NK Cells

Natural Killer Cells (NK cells) are type of innate lymphocytes mediates anti-viral and anti-tumor activity. NK cells develop in the bone marrow (BM) and secondary lymphoid tissues such as, tonsils, spleen and lymph nodes (LNs) and they represent 5-20% of circulating lymphocytes in humans (77, 78). NK cells are distinguished from the other immune cells by possessing CD3^-^ and CD56^+^ phenotype. Human NK cell subsets express also CD16 molecule, which is involved in antibody dependent cellular cytotoxicity (ADCC). NK cells are effector cytotoxic cells, they recognize and destroy their target without prior sensitization. Unlike T cells, they do not need MHC class presentation to enact their cytotoxic properties. Unlike T cells, NK cells recognize and kill tumor in an HLA-independent manner which result in being known as a great candidate for allogeneic anti-tumor cell-based therapies, as they do not cause acute GvHD (79–81). NK cells use KIR receptor and ligand mismatch to recognize cancer cells from self-cells, therefore mediating enhanced engraftment, anti-tumor response, and safe clinical outcomes (79, 81–85). NK cell killing of target cells accomplished with a balance of activating and inhibitory signals engaged around the cell. NK cell activating receptors includes, killer cell’s immunoglobulin-like receptors (KIRs), KIR2DS2, KIR2DS5 KIR3DS1, CD94/NKG2C, NKG2D, NKp30 NKp40, NKp44 and NKp46 recognize ligands present on target cells. NK cells have the ability of recognize non-self by NKp80, SLAM, CD18, CD2 and TLR3/9 receptors. Some of the NK cell inhibitory ligands are PD-1, TIGIT, TIM-3 and LAG-3. Inhibitory KIR ligands, KIR2DL1, 2DL2, and 2DL3 interact with highly polymorphic human leukocyte antigen (HLA). There are three HLA groups, group 1, 2 and HLA-Bw4, which usually bind inhibitory KIR and have long extracellular immunoglobulin structure. It has been shown that patients who receive NK cell immunotherapy containing haplo-mismatched NK cells they have anti-leukemic effects without the risk of GVHD. In hematopoietic stem cell transplant (HSCT) patients, infusions of haplo-mismatched KIR and HLA NK cells has shown benefits of survival and lower relapse rates. If the infused NK cells are identical, they only show benefit if the KIR receptors are activating (86, 87). NK cells can be isolated from peripheral blood, umbilical cord, and induced pluripotent stem cells (iPSCs) (88–91). Once isolated from their primary source, feeder cells, such membrane-bound IL-21 K562s, used to expand NK cells ex-vivo (92). They can be cultured anywhere from 14-21 days in most protocols and can proliferate remarkably over hundreds of folds (92). Cytokines such as IL-2, IL-12, IL-15, IL-18, and IL-21 are also added in NK cell cultures to enhance NK cell proliferation and activation (86, 87). NK cells have several mechanisms to eradicate their targets. One of the main mechanisms is perforin and granzyme induced apoptosis. Granzymes which are serine/proteases, packaged along with perforin and when they release by NK cells, they initiate target apoptosis *via* caspase-3 pathway. In addition to that, NK cells *via* Fas ligand and tumor necrosis factor (TNF)-related apoptosis-inducing ligand (TRAIL) pathways can destroy their targets (93–95).

## Examples of CRISPR Edited NK Cells

CRISPR editing of NK cells has been challenging, however we and others have shown that using electroporation of Cas9/RNP can solve the issue of low viral transduction efficiency of NK cells (18, 23, 96–103). Gene editing in NK cells in a short period since its invention has been used for serval applications such as to improve their metabolic function, knocking-out checkpoint molecules, improving antibody therapies and generation of CAR-NK (96). One great example of gene engineered NK cells is CD38 knock-out NK cells. NK cells highly express CD38 on their surface. Patients treated with daratumumab (Dara, hereafter), a monoclonal antibody targeting CD38 on multiple myeloma, showed a decrease in NK cells number. This is a result of NK-NK recognition through CD16 biding to Dara coated CD38+ NK cells, referred to as “fratricide.” Beyond the role of the structural marker, CD38 is well described to be associated with a large diversity of physiological and pathological conditions. Our group and others successfully developed NK cells lacking CD38 by introducing the CRISPR/Cas9 as Cas9/RNP *via* electroporation (96, 101). In particular, CD38 is an NAD-degradation enzyme in mammalian tissues (104–110). Our data demonstrated that CD38KO NK cells have more prominent metabolic profile, increased killing mediated by ADCC against CD38^+^ multiple myeloma cell lines and patient derived samples and are protected from fratricide mediated by daratumumab (96, 101).

Another important target to improve the NK cell’s function is CISH encoded by CIS gene. CISH has a critical impact on NK cells, and its activation is known to disable JAK-STAT downstream signaling pathways including a decline in NK cell ability to kill malignant cells (111, 112). Different groups have shown that CISH is overexpressed in the presence of IL-2 and IL-15 (113–115). IL15 was previously described as an important factor potentiating NK cells cytokine production and cytotoxicity activity (116–118). Felices et al. have demonstrated that prolonged administration of IL15 can unleash NK cells exhaustion *via* metabolic failure (119). Delconte et al. showed that *CISH* was quickly activated after IL15 stimulation in a mouse model, supporting that using gene-editing in NK cells to delete CISH seems to be advantageous (120). Using CRISPR/Cas9 on human iPSC to generate iPSC-CISH knockout NK cells displayed prolonged persistence *in vivo* and enhanced antitumor activity for acute myeloid leukemia (121, 122). NK cell checkpoint blockade has been used as a promising therapy for liquid and solid tumors. Other candidate for gene editing in NK cells is NKG2A which is an immune checkpoint in CD8+ αβ T cells, natural killer T cells (NKT) and CD56hi NK cells. Upon activation of immune cells, NKG2A leads to decreased effector function (123, 124). Data from the literature have shown that NKG2A drives NK cells to fatigue when highly expressed, and it can be predictive of poor prognosis in liver cancer patients (125). Thus, the blockage of the NKG2A receptor enhances NK cell’s effector function for immunotherapy (126–128). Similarly, Berrien-Elliot et al., have shown that gene-editing using CRISPR/CAS9 to delete NKG2A from human NK cells was able to increase NK cell ability to control HLA-E^+^ K562 leukemia when compared to control NK cells demonstrating a substantial inhibitory function for NKG2A (129). Additionally, NKG2A^KO^ NK cells did not affect their persistence in NSG mouse model (129), however, the role of NKG2A in NK cells licensing may cause development of unlicensed NK cells with lower cytotoxic activity (130). It is very well established that the PD1/PD-L1 axis has an inhibitory function that can impair many T cells’ functions. This fact has been validated in preclinical models where the inhibition of this signaling cascade is used for cancer treatment (131). Indeed, high expression of PD1 ligand I or II in cancer cell lines impairs cytotoxic function on CD8+ T cells. On the other hand, the absence of a functional PD1 was responsible for tumors rejection in the murine model (132, 133). The blockage of the PD1/PD-L1 axis with monoclonal antibodies repair these effects and unleash T cells to effectively kill tumor cells (132–134). Recently it has been shown that in different malignancies, human NK cells also express PD-1 (135–139). Like T cells, blockade of the PD1/PD-L1 axis was able to activate NK response (140). However, such strategies present limitations, especially regarding off-target toxicity (102). Pomeroy et al. could generate PD1^KO^ NK cells by electroporating mRNA Cas9 and gRNA (102). They demonstrated that PD1^KO^ NK cells showed notably enhanced cytotoxicity and cytokine secretion *in vitro* and *in vivo*, decreasing tumor burden that culminated with survival (102). Another promising target for gene editing to boost cancer immunotherapy is the Suppressor of cytokine signaling 3 (SOCS3). The protein SOCS3 is one among eight members of the Suppressor of cytokine signaling family (SOCS1–7 and CIS). Those proteins downregulate cytokine signaling *via* the JAK/STAT signaling cascade. Murine NK cells upregulated SOCS3 expression after IL-15 stimulation (120). SOCS3 impair inflammation by inhibiting pro-inflammatory signaling pathways, including IL-12 inducing IL-12Rβ2 subunit blockage *via* the SH2 domain and its signaling pathway mediated by STAT4 (122). The absence of SOCS3 does not impact NK cells function upon IL15 stimulation in murine models. In humans NK cells, our group successfully generated SOCS3^KO^ NK cells using Cas9/RNP and showed higher cell proliferation and enhanced NK cells anti-tumor activity (100). Suggesting SOCS3^KO^ NK cells could be an excellent target for gene-editing to boost cancer immunotherapy. Another novel target is ADAM17, this gene has well described as a membrane-associated protease responsible for cleaving a large variety of membrane molecules, including CD16 (102, 141–144). Blocking ADAM17 activity leads to improvement in cytokine production of human NK cells due to maintaining their CD16 on the cell surface and activating higher ADCC when combined with antibodies (145). Pomeroy et al. have demonstrated that CRISPR-edited ADAM17^KO^ NK cells are prevented against CD16 shedding compared to WT NK cells (102). Additionally, those data are similar to ADAM17 inhibitors where treated groups presented enhanced killing through ADCC. Similarly, Yamamoto et al. showed that ADAM17 gene-edited iPSCs derived NK cells have enhanced ADCC (102, 141, 144–146).

**Figure 2:**
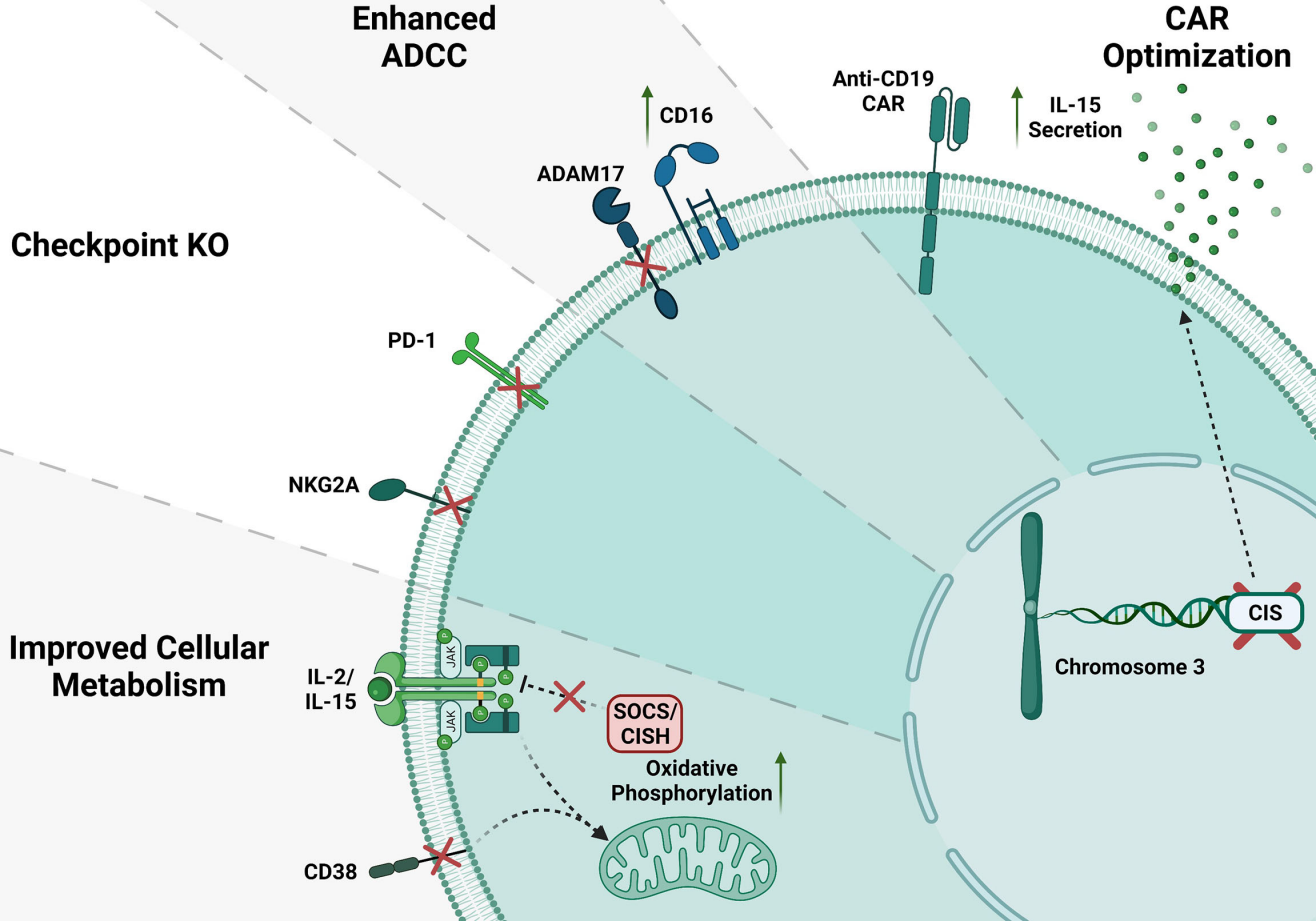
CRISPR gene editing in NK cells. Several gene KO in NK cells have been done to improve NK cell function; here, we show some of the NK cell gene modifications. CD38 and SOCS/CISH KO can improve metabolism in NK cells. Inhibitory checkpoint receptor KO such as NKG2A and PD-1 KO. ADAM17 KO enhance CD16 mediated ADCC. Anti-CD19 CAR NK cells increase IL-15 production and enhance NK cell anti-tumor activity.

To improve immune cell recognition and killing towards tumor cells, immune cells, including T cells and NK cells are engineered to express chimeric antigen receptors (CARs) (147–149). In one of the first clinical trials using iPSC CD19-CAR NK cells, the patients treated with the CAR-expressing NK cells showed some improvements in their clinical outcomes (150). Generation of CAR-NK cells have been challenging due to the low efficient viral transduction including CAR-NK cells used in the trial mentioned above. Our group recently showed that we could efficiently combine Cas9/RNP approach with self-complementary (sc) Adeno-associated virus (AAV) or single-stranded gene delivery for generating highly efficient human primary CAR-NK cells (98). Using this approach, we developed CD33 CAR-NK cells (98). These CAR-NK were efficiently able to kill AML cells and showed improvement on their activation markers (98). Similar data were obtained when CD33-CARNK cells co-culture with patient samples (97, 98). Recently, Daher et al. showed that CRISPR edited CIS-KO NK cells expressing CAR-IL-15 construct could boost CAR-NK cell function *in vitro* and xenograft models by increasing aerobic glycolysis (121). This double enhancement of CAR-IL-15/CIS-KO signaling is significantly beneficial in the TME (151). Overall, gene editing of NK cells has been challenging but the recent successes in using CRISRP by electroporating Cas9/RNP helped to improve the outcome of the NK cells therapy (**Figure 2**) (18, 101, 103, 152, 153). There has been some evidence showing that Polymer-stabilized Cas9 nanoparticles and modified repair templates can increase genome editing efficiency. These modified nanoparticles improved knock-out and knock-in efficiency of the CRISPR gene editing in several primary cells such as NK and T cells (16).

### Clinical Trials Using CRISPR Edited NK Cells and T Cells

Advancements in immunotherapy and gene therapy opened a new era for clinical trials to treat some hematological malignancies and solid tumors. Along with other platforms, CRISPR/Cas9 technology was adapted and brought up to the clinic to correct some mutations and boost immune responses. CRISPR/Cas9, as a precise gene-editing tool with minimal cytotoxicity and off-target effects, has become a promising approach to treat complex and refractory diseases. However, due to some limitations, including transduction efficiency, off-target mutations, ethical questions, and the deficiency in scientific risk assessment, CRISPR/Cas9 gene-editing clinical trials have not been prevalent, especially for T and NK cells. However, CRISPR has opened its way to the clinic. One of the first in human phase 1 clinical trial of using CRISPR engineered T cell have been used for patients with refractory cancers in the U.S. (clinicaltrials.gov; trial NCT03399448) (68). In this trial, endogenous TCR and immune checkpoint molecule PD-1 were targeted in T cells with CRISPR/Cas9 to improve immunotherapy in several refractory cancers. Two patients with advanced refractory myeloma and one with metastatic sarcoma were treated with these CRISPR-edited cells (68). The results of this trial demonstrated the safety of infusing CRISPR-edited ex-vivo expanded CAR-T cells in patients (151). Examples of some clinical trials with the CRISPR/Cas9 method in T cells are presented in **Table 1**. However, there are no registered CRISPR/Cas9 transduced CAR-NK cell clinical trials in the United States.

**Table 1:** Examples of clinical trials with CRISPR/Cas9 gene edited T cells (151).

National Clinical Trial Number	Cancer	CRISPR target gene	T cell source	Technique	Country
NCT04037566	Relapsed or refractory ALL and B-cell lymphoma	HPK1	Autologous T cells	Rnp Electroporation	China
NCT03399448	Multiple myeloma, melanoma, synovial sarcoma, myxoid/round cell liposarcoma	TCRα, TCRβ and PD-1	Autologous T cells	Rnp Electroporation	USA
NCT03545815	Solid tumors	Endogenous TCR and PD-1	T cells (unknown source)	N/A	China
NCT04244656	Refractory multiple myeloma	B2M gene and TCR	Allogeneic T cells	N/A	USA and Australia
NCT03747965	Solid tumors	PD-1	T cells (unknown source)	N/A	China
NCT04035434	B-cell malignancies	B2M gene and TCR	Allogeneic T cells	N/A	USA and Australia
NCT03166878	B-cell leukemia and lymphoma	B2M gene and TCR	Allogeneic T cells	Rnp Electroporation	China
NCT03044743	EBV related diseases	PD-1	EBV CTL from autologous source	N/A	China

N/A stands for non-applicable.

## Off-Target Analysis of CRISPR Edited Immune Cells

Recently by the promises of Cas9 endonuclease, researchers can target multiple genes in immune cells, including T cells and Natural killer (NK), to improve cancer immunotherapy. For these applications that lead to clinical cancer immunotherapy, the induced mutations by CRISPR-Cas9 should be highly precise and specific for the targeted loci with high on-target efficiency and low or no off-target activity. However, rare off-target events are inescapable during the manipulation of the gene of interest. This phenomenon requires scrutiny identification, especially in clinical applications to cure cancers and avoid adverse effects during cancer immunotherapy such as introduction of an oncogene. By developing next-generation sequencing (NGS) a survey of new functional and non-functional variations during gene manipulation became possible (154, 155). NGS has been broadly applied by researchers and employed in clinical trials due to its development in data acquisition with speedy and high-quality recognition (156, 157). Analyzing these NGS-generated data is even more critical to optimize and manage the workflow to fill the gap between massive data and scientific exploration. To date, several methods have been invented to analyze NGS data and off-target effects of CRISPR mediated mutations, such as GUIDE-seq, SITE-Seq, CHANGE-seq, Cas-OFFinder and Churchill (158–162). Some of them like GUIDE-seq, SITE-Seq and CHANGE-seq are based on the PCR amplification of pre-selected potential sites, which predicted by CRISPR/Cas9 design tools, and sequencing the PCR amplicons utilizing Sanger or NGS technologies (158–160, 163). For instance, Schumann et al., used a 2-step PCR method and sequenced with the amplicons with Illumina HiSeq, and identified indel mutations and their spatiality distribution in the target region in primary human T cells (75). In another study the efficiency and indel rates in the created CAR-T cells, using CRISPR-Cas9-mediated multiplex gene editing, was quantified by both surveyor assay and tracking of indels by decomposition (TIDE) analysis (58). Stadtmauer et al. utilized iGUIDE, a modified method of GUIDE-seq, for the Cas9-mediated cleavage specificity analysis in the engineered T cells to cure refractory cancer and found no clinical toxicities (68, 158, 164). Although these methods are simple and available to most molecular biology laboratories, they are not always precise as they are based on the predictions of potential off-target sites by CRISPR/Cas9 design tools in the genome of interest and therefore result in studying limited loci. As a matter of fact, DSBs happened beyond the predicted sites and may be ignored and caused detrimental side effects during the process of clinical cancer immunotherapy (163). This major disadvantage of off-target mutations identification by PCR based methods have been resolved by whole genome sequencing (WGS) which is unbiased and has been used to screen for off-target mutations induced by CRISPR/Cas9 in different cells including human inducible pluripotent stem cells, primary T cells, CAR-T cells (163, 165–167). Using this method, researchers can recognize both small indels and SNPs as well as major deletions, inversions, duplications and, rearrangements (163, 166). The only restriction of whole genome sequencing is missing the most low-frequent off-targets that happens to a small number of clones (163, 168). Cas-OFFinder algorithm have been invented in order to search for potential off-target sites in any sequenced genome regions (161). In a clinical trial, the safety and feasibility of CRISPR–Cas9 PD-1-edited T cells were confirmed after analyzing all the potential off-targets using Cas-OFFinder method in the treatment of lung cancer (169). More recently, as an ultra-fast, definite, highly scalable, and balanced parallelization strategy for discovering human genetic variation in clinical and population-scale genomics, Churchill has been applied for the analysis of next-generation sequencing data (162). We reported the high efficacy of Churchill analysis in verifying off-target events after deletion of CD38 in NK cells *via* Cas9/RNP and showed low off-target effects of Cas9/RNP (96). It has successfully revealed all the existing mutations and categorized them as missense and non-frameshift and moderate or high impact (96). Overall, WGS can provide more precise landscape of the off-target effects in CRISPR-edited cells. Here, we summarize and compare the current methods in off-target effects analyses of CRIPR edited immune cells (**Table 2**).

**Table 2:** Current methods in off-target analyses of CRISPR edited immune cells.

Off target analysis method	Definition	Pros	Cons
Cas-OFFinder (161)	It is an algorithm that searches for possible off-target sites that can be found in an already sequenced genome.	- It is not limited by the number of mismatches and the PAM sequence. - It allows alterations in PAM sequences which are differentiable with Cas9. - a rapid and highly assorted off-target searching tool available at http://www.rgenome.net/cas-offinder	- it relies on a computational method, which may result in ignoring some potential off-targets sites. - it is biased due to the assumption that off-target sequences are affiliated with the on-target site which may cause missing off-target sites in any loci throughout the genome.
SITE-Seq (selective enrichment and identification of tagged genomic DNA ends by sequencing) (159)	It is a biochemical method, using Cas9 and single-guide RNAs (sgRNAs), to recognize all the Cas9-mediated cut site sequences inside the genomic DNA.	- It allows retrieval of off-target sites with different cleavage sensitivity by utilizing a vast range of sgRNP concentrations from very low to high. - Provides guidance for precise and plenary inspection of possible off-target sites in cells by gaging the incidence of mutations and their functional cellular effects. - Production of sequencing libraries which are highly enriched for sgRNP cut sites, providing unique profiling with minimal read depth.	- DNA-repair machinery does not have a role in the process as it is performed on high molecular weight DNA.
GUIDE-seq (genome-wide, unbiased identification of DSBs enabled by sequencing) (158)	It is a PCR-based method that relies on the enteral of double-stranded oligodeoxynucleotides into the DSB caused by RNA-guided nucleases (RGN) without contributing to off-target site.	- Enables to turn out universal specificity perspective for different RGNs - Identifies the hotspots in DNA breakpoints that can take part together with RGN-induced DSBs in higher-level genomic alterations such as translocations. - Its performance on living cells enables capturing of DSBs that occur over a more extended period, thereby making it a more delicate and plenary assay.	-Relies on an integration of donor sequences, which usually happens in a low frequency. - mispriming may occur due to the annealing of PCR primers to DNA sequences apart from the ODN, resulting in PCR products that are not differentiable from products formed by primers binding to the ODN.
iGUIDE (improvement of the GUIDE-seq method) (164)	GUIDE-seq method allows mis priming artifacts to be recognizable from credible ODN integration sites by using a larger ODN (46 nt versus 34 nt).	- by using larger ODN, PCR primer binding sites can be back off from the junction of the ODN in the final PCR product and can cause mis priming events.	-It is tough to scale due to individual transfections for each target or cell source.
ChIP-seq (chromatin immunoprecipitation sequencing) (158)	It identifies the off-target binding sites by using catalytically dead Cas9 (dCas9)-gRNAs complex.	- Important for the identification of the genome-wide binding sites with dCas9 fusion proteins.	-It rarely indicates the off-target sites of cleavage caused by active Cas9 nuclease. -not effective for recognition of genome-wide, off-target cleavage sites for catalytically active RGNs. -cost and availability
CHANGE-seq (circularization for high-throughput analysis of nuclease genome-wide effects by sequencing) (160)	It is a high-throughput procedure for determining the genome-wide operations of CRISPR-Cas9 nucleases based on Tn5 mediated gDNA tagmentation *in vitro*.	- A simplified, susceptible, and scalable approach. - It can elucidate the genome-wide perspective of genome editing activity exquisitely sensitive. - elaborated to efficiently procreate circularized genomic DNA libraries for elucidating the genome-wide activity of genome editors by leveraging a new Tn5 tag mentation-based workflow.	-it relies on the Tn5 tagmentation of donor sequences. - Similar to SITE-Seq, the DNA repair machinery is ignored.
Churchill (162)	In clinical and population-scale genomics provides fast, decisive, scalable, and balanced parallelization tactic for the detection of human genetic mutation.	- It uses a robust comparison based on whole genome sequencing data comparing wildtype and CRISPR edited cells. - The procedure is highly scalable, authorizing full resolution of the 1000 Genomes raw sequence dataset utilizing cloud resources in a week. - It eliminates the bottlenecks of the computational sequence analysis impasse *via* the avail of cloud computing resources. - It matches with the amplitude of genomic data.	- Limited access to the platform and the algorithm is not publicly available yet.

## CRISPR Screening in Primary Immune Cells

Genome wide CRISPR screen has been used in several cancer cells to discover novel targets for cancer immunotherapy. CRISPR screening approach has not been extensively used in human primary immune cells due to several technical challenges. However, some studies have shown successful screening approaches in human primary T cells and Cas9-expressing transgenic mice in recent years (19, 170–172).. In general, to perform a CRISPR screen we need to introduce Cas9 and gRNA pool library into the cells (173). These molecules usually delivered to the target cells *via* lentiviral transduction. However, expressing large proteins such as cas9 using LV vectors in immune cells such as NK cells and T-cells has been challenging and results in low transduction efficiency. Shifrut et al; tested a hybrid approaching which the Cas9 was introduced to the gRNA library expressing cells *via* electroporation (19). They developed Single guide RNA (sgRNA) lentiviral infection with Cas9 protein electroporation (SLICE) and resulted in discovery of novel genes important in activation and expansion of CD8 T-cells (19). A similar approach was used by other groups to perform CRISPR screening in CAR-T cells (174). To date, there is no publication on CRISPR-screening on NK cells. Our group is investigating some new approaches to overcome issues related to lentiviral transduction of NK cells.

## Conclusion

CRISPR gene editing technology has shown to be a very versatile tool for improving anti-tumor activity of NK cells and T-cells. We reviewed here some of the CRISPR edited cells used for cancer immunotherapy. We also reviewed ways to determine the off-target effects of CRISPR and emphasized that Cas9/RNP approach results in low off-target effects. We also mentioned how important information can be discovered by CRISPR screening approach and there are a lot to do the efficiently optimize this method to be used in NK cells and T cells. Overall, CRISPR gene editing shows promising clinical outcome and have potentials to be used more broad Clinical applications such as cancer immunotherapy using NK cells and T cells.

## Author Contributions

The corresponding authors MNK and DAL supervised the authors for manuscript completion. The first author EE contributed to manuscript writing and revising. All authors contributed to the article and approved the submitted version.

## Conflict of Interest

MN reports personal fees from Kiadis Pharma; in addition, MN has patents US62/825,007; WO2019222503A1; USPTO63/105,722; PCT/US2020/02545; US63/018,108; US62/928,524; US62/987,935; self-driving CAR with royalties paid by Kiadis Pharma. DL reports stock from Courier Therapeutics, personal fees and stock options from Caribou Biosciences, personal fees from Intellia Therapeutics, personal fees from Merck, Sharp, and Dohme, grants, stock, and personal fees from Kiadis Pharma, outside the submitted work; in addition, DL has patents US62/825,007; US63/105,722; US62928,524; PCT-US201/032,670; WO-2019/222,503-A1; PCT-US2020/018,384; US62/805,394; US62/987,935; US62/900,245; US62/815,625; Self-driving CAR with royalties paid to Kiadis Pharma and Membership on the NIH Novel and Exception Therapies and Research Advisory Committee (NExTRAC). MSFP reports stocks from MERCK, Fate Therapeutics, Sorrento Therapeutics, Moderna and received licensing fee from Kiadis Pharma.

The remaining authors declare that the research was conducted in the absence of any commercial or financial relationships that could be construed as a potential conflict of interest.

## Publisher’s Note

All claims expressed in this article are solely those of the authors and do not necessarily represent those of their affiliated organizations, or those of the publisher, the editors and the reviewers. Any product that may be evaluated in this article, or claim that may be made by its manufacturer, is not guaranteed or endorsed by the publisher.
